# Work–Life Balance Does Not Mean an Equal Balance

**DOI:** 10.3389/fped.2016.00018

**Published:** 2016-03-14

**Authors:** Mayte Figueroa

**Affiliations:** ^1^Division of Cardiology and Critical Care, Department of Pediatrics, Washington University School of Medicine, St. Louis, MO, USA

**Keywords:** work, life balance, gender differences, leadership, medical marriage

As I reflect on the different phases of my life as a physician, wife, mother, and physician leader, I realize that my expectations or definition of work–life balance have varied. I might even dare to say that my expectations vary on a daily basis. The concepts that remain at the crux of what I consider most important are a feeling of daily achievement and joy in each of my four life quadrants. These consist of Career, Family, Friends, and Self. At each stage of my life, I have challenged myself with changes that could potentially have caused an imbalance but instead increased my sense of self achievement and enjoyment. Literature has shown that female leadership in medicine is still disproportionately small which might be due to the barriers of combining work and family (1, 2). Compared with the early 1950s, today the number of women and men who successfully finish medical school is approximately equal. Despite this, a publication by Non-nemaker in the New England Journal of Medicine in 2000 showed that women who enter academic medicine have been less likely than men to be promoted or to serve in leadership positions (3). Some of the individual barriers to career development include the sporadic focus on career advancement, time-consuming child care, family responsibilities, and a woman’s tendency toward understatement. Despite these barriers, work–family enrichment has been shown to have a positive spillover effect that spreads positive energy and helps to balance the work–life relationship (4). My communication, teamwork, and leadership skills influence my work and home environments in a positive way. Within my marriage, there is a mutual support that we both rely on as well as recognition of the important role each member plays. If asked, what would we say our strategies for success are in a two career family? First, having a set time for synchronizing schedules; second, frequent verbal support; and third, shared decision making (5). Other strategies that have been reported to play an important role in the medical marriage include defining and recognizing the important roles of each family member (6). For example, determining who does certain chores pays the bills or carpools, it is important to have clarity of our own and our partner’s responsibilities. Having shared values with a spouse/partner really defines the foundation of a marriage/relationship and serves as a frame of reference when competing commitments arise or when faced with challenges and difficult issues.

Family life in the United States has changed. A recent survey reported that most dual-earning families include a parent working long hours at atypical times (7). Academic medicine can develop so that it supports family life and retains women, but there are several steps that must be taken. First, we must have realistic expectations of what one can accomplish in a day. For me, family time is valuable and my daughter and spouse are not a hindrance or burden to academic or clinical medicine, they are what grounds me in the real world. Second, we need to foster the right kind of mentoring. A mentor should be one who appreciates the things that make our lives work. Third, we need to develop collaborative links between women to support and learn from each other through coaching and networking. Most barriers to career progression are shared. We are not alone in feeling undervalued and overwhelmed.

The Institute of Medicine’s landmark publication, “Beyond Bias and Barriers: fulfilling the potential of Women in Academic Science and Engineering,” explored why women are underrepresented in academic medicine (8). Their conclusion was that women’s underrepresentation was due to a steady attrition of women throughout their careers rather than a shortage of women entering these fields. Data from AAMC benchmarking surveys indicate that a number of medical schools already have programs that support the professional development of female faculty, but the nature of such support varies substantially (9). Successful programs should provide an inclusive and supportive climate and unique opportunities for female faculty to network, interact, and collaborate with each other.

Lastly, coming to the realization that work–life balance does not mean an equal balance will make life more realistic and rewarding. We should not have to place our different roles at odds with each other competing for time. Instead of desiring work/life balance perhaps one should seek inner happiness.

This instead should be our measure of success.

## Author Contributions

MF, MD FACC Associate Professor of Pediatrics at Washington University School of Medicine and St. Louis Children’s Hospital. Only author on this opinion article. Performed background literature search and prepared manuscript.

## Conflict of Interest Statement

The author declares that the research was conducted in the absence of any commercial or financial relationships that could be construed as a potential conflict of interest.
